# Congenital toxoplasmosis in a newborn of a chronically infected mother

**DOI:** 10.1590/S1678-9946202668047

**Published:** 2026-07-24

**Authors:** Vitória Schneider Müller, Júlia Sales Machado, Wilson Hoshino, Aida de Fátima Tomé Barbosa Gouvêa, Fabiana Bononi do Carmo, Kelly Simone Almeida Cunegundes, Maria Aparecida Ferrarini, Annelise Pereira Barreto Monteiro, Daisy Maria Machado, Ana Isabel Melo Pereira Monteiro

**Affiliations:** 1 Universidade Federal de São Paulo, Escola Paulista de Medicina, São Paulo, São Paulo, Brazil; 2 Universidade Santo Amaro, São Paulo, São Paulo, Brazil

**Keywords:** Congenital toxoplasmosis, Pregnancy, Reinfection, Vertical transmission, Reactivation

## Abstract

Congenital toxoplasmosis rarely occurs in infants born to mothers with evidence of prior immunity. We report the case of a preterm infant with severe ocular and neurological manifestations of congenital toxoplasmosis, despite maternal seropositivity (IgG+/IgM–) during pregnancy. This case highlights the potential for reactivation or reinfection with virulent strains and reinforces the need for vigilance and preventive strategies throughout pregnancy.

## INTRODUCTION

Toxoplasmosis, caused by *Toxoplasma gondii*, remains a relevant public health issue, especially during pregnancy due to the risk of congenital infection. Transmission occurs mainly via ingestion of undercooked meat or contaminated water, fruits, or vegetables, while vertical transmission results from maternal parasitemia during gestation. In South America, particularly in Brazil, prevalence of IgG antibodies is high, and more severe outcomes have been reported due to the genetic diversity and virulence of circulating strains^1^. Although routine screening during pregnancy is mandatory and immunity is defined by positive IgG and negative IgM, congenital toxoplasmosis may still occur in mothers with prior immunity^2^. Cases of vertical transmission have been associated with reactivation during gestational immunosuppression or reinfection with different or more virulent strains^3-5^.

We report a case of congenital toxoplasmosis in a newborn born to a previously immune mother, underscoring the need for clinical vigilance and comprehensive preventive strategies throughout pregnancy.

### Ethics

This case report was approved by the Ethics Committee of the Universidade Federal de Sao Paulo (UNIFESP), under protocol Nº CAAE 68844723.5.0000.5505. Written informed consent for publication was obtained from the child's legal guardian.

## CASE REPORT

A female infant of mixed-ethnicity, from Sao Paulo city, Brazil, was born in 2021 at 33 weeks of gestation by cesarean section due to acute fetal distress. The mother was 35 years old, multiparous with four pregnancies, three live births, and one abortion, and initiated prenatal care at 10 weeks. Her pregnancy was considered high risk because of gestational diabetes requiring insulin therapy from 24 weeks of gestation onward.

Maternal serologic tests performed in 2019 showed IgG of 64.1 U/mL and IgM of 0.09 U/mL by chemiluminescence essay. The patient provided prenatal laboratory results, records of previous hospitalizations, and test results from prior pregnancies. She did not receive specific treatment after testing positive for toxoplasmosis. During the current pregnancy, IgG-positive/IgM-negative serology was recorded on June 17, 2021 (ninth gestational week). Near delivery (December 22, 2021), serology showed IgG of 200 U/mL, IgM <0.5 (non-reactive), and IgG avidity of 99%, also by chemiluminescence essay. No serial serologic monitoring for toxoplasmosis was performed during pregnancy. Other tests were negative for HIV, hepatitis B and C, and syphilis, while cytomegalovirus serology showed reactive IgG and non-reactive IgM. The mother underwent ophthalmologic evaluation only during the postpartum period, and fundoscopic findings were normal. Originally from Bahia State, the mother had been living in Sao Paulo for six years in a brick house with access to sanitation and electricity. She reported frequent exposure to soil and cat excrement while maintaining the property, which also housed several cats.

The newborn weighed 1,430 g at birth and was admitted to the neonatal intensive care unit (NICU) due to respiratory dysfunction, requiring oxygen therapy in an incubator, as well as phototherapy for six days. Ocular screening revealed bilateral absence of the red reflex. The heel prick test was performed at three days of life and was positive for toxoplasmosis (IgM+). Neonatal serology at 13 days of life showed IgG of 200 U/mL and reactive IgM of 5.2 U/mL. Initial cranial ultrasonography findings were normal.

At one month of age, ophthalmologic evaluation diagnosed bilateral chorioretinitis. A subsequent cranial ultrasound revealed small cerebral parenchymal calcifications. Treatment was initiated with sulfa­methoxazole/trimethoprim, pyrimethamine, folinic acid, and prednisolone. According to the mother, the infant initially received sulfamethoxazole/trimethoprim because sulfadiazine was temporarily unavailable in the public health system. We had access to the newborn's full medical record at our institution from two months of age onward. Earlier information was retrieved from the discharge summary of the referring facility. At the first visit to our clinic, the standard regimen consisting of sulfadiazine (100 mg/kg/day), pyrimethamine (1 mg/kg/day), folinic acid (45 mg/week), and prednisolone (1 mg/kg/day) was initiated, with prednisolone tapered after the fourth month.

At six months of age, the patient developed neutropenia (absolute neutrophil count: 752 cells/mm^3^), leading to reduction of pyrimethamine administration to three times weekly, with subsequent recovery of neutrophil counts. Overall, treatment was well tolerated, and the regimen was maintained until 13 months of age.

**Figure 1:**
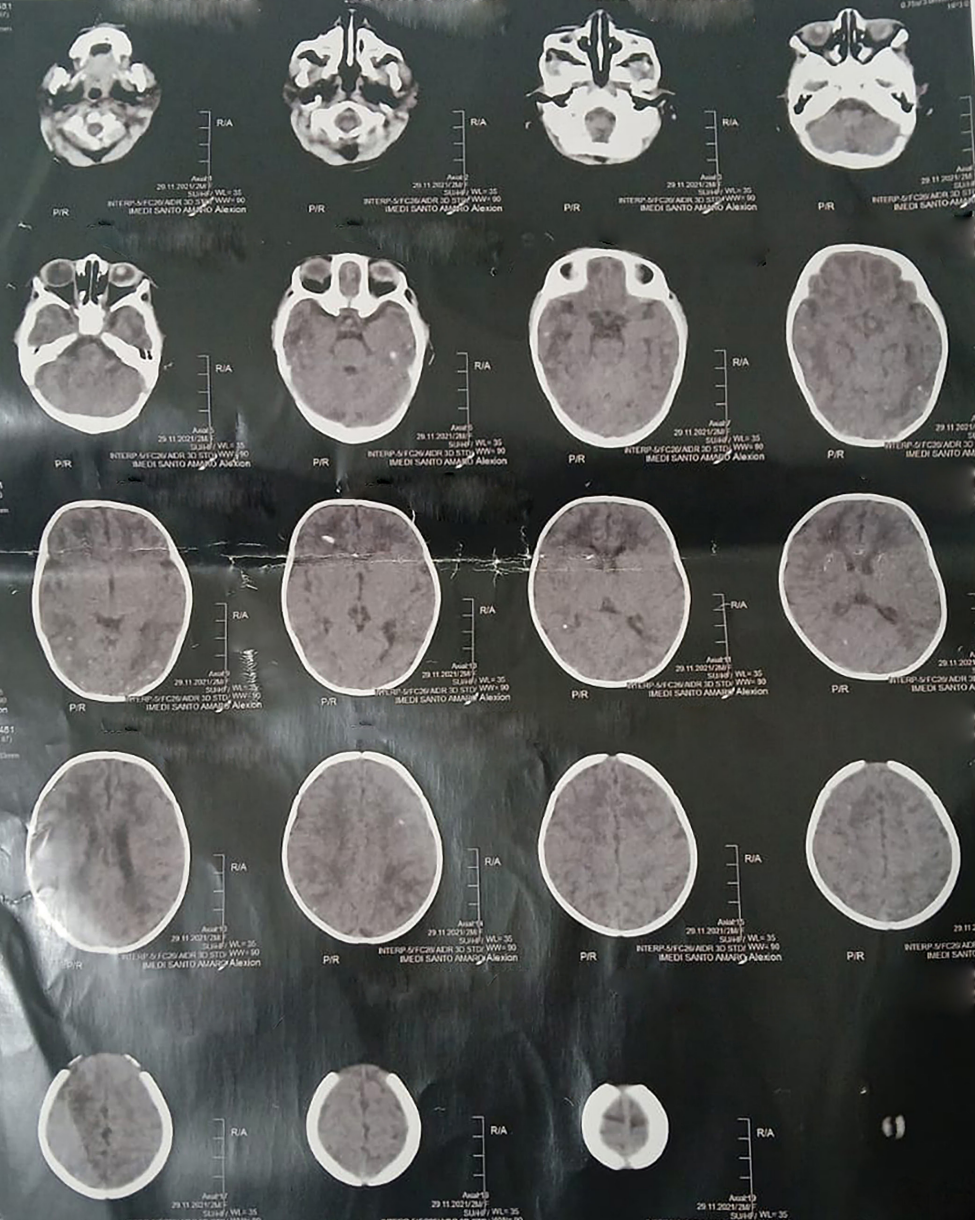
Cranial CT scan revealed small cerebral parenchymal calcifications.

Serial ophthalmologic evaluations showed bilateral chorioretinal scars, vitreous opacities, posterior synechiae, and pale optic nerves. Horizontal nystagmus was observed; the right eye tracked objects without fixation, while the left eye responded only to light. Cranial computed tomography (CT) performed at three months of age revealed bilateral calcifications and white matter hypoattenuation (Figure 1). Further magnetic resonance imaging (MRI) and electroencephalography (EEG) are pending. Brainstem auditory evoked potentials were normal.

The diagnosis of congenital toxoplasmosis was based on: (1) characteristic neuroimaging findings; (2) bilateral active chorioretinitis; (3) reactive neonatal IgM detected at 13 days of life (beyond the 10th day), associated with elevated IgG titers; and (4) positive newborn screening results.

At present, the child remains under ophthalmologic and neurologic follow-up. She uses corrective lenses because of visual impairment secondary to chorioretinitis; however, despite significant ocular sequelae, her neuropsychomotor development remains within normal limits.

## DISCUSSION

This case highlights the possibility of vertical transmission of *Toxoplasma gondii* in pregnant women previously considered immune, raising concerns about the limitations of acquired immunity. Although routine serologic findings of IgG+/IgM– are generally interpreted as protective, our findings add to the growing evidence that reactivation or reinfection may occur during pregnancy, especially in regions where virulent strains circulate, such as South America.

Ocular and neurological manifestations remain the most frequent and severe sequelae of congenital toxoplasmosis, as observed in this patient, who developed bilateral chorioretinitis and cerebral calcifications. These findings are consistent with previous reports that emphasize the burden of ocular disease in congenital cases and its impact on long-term prognosis^3-5^.

Recent studies, including reports from Brazil, have confirmed that infants may be born with severe manifestations despite maternal serology compatible with past infection^6^. Such cases reinforce the hypothesis that protective immunity may be strain-dependent and that gestational immunosuppression can favor parasite reactivation. Emerging evidence also suggest that gestational diabetes mellitus (GDM) may impair both humoral and cellular immune responses, even in chronically infected women. In a study by Oliveira-Scussel *et al*.^7^, pregnant women with GDM chronically infected with *T. gondii* showed reduced IgG avidity, lower production of Th1/Th2/Th17 cytokines, and diminished T-cell activation compared to non-GDM controls. Such findings support the possibility that GDM may compromise the protective immunity against *T. gondii* and favor vertical transmission. Therefore, counseling should be provided to all pregnant women, regardless of serological status.

These findings are consistent with Brazilian pediatric guidelines, which recommend continuous surveillance and preventive measures to reduce the burden of congenital toxoplasmosis^8^. This case also reinforces the importance of newborn screening programs (heel prick test) for early detection of congenital toxoplasmosis, especially in settings where maternal immunity may not prevent fetal infection. In addition to prenatal surveillance, exposed newborns require structured postnatal follow-up with serial clinical and serologic evaluation, even when maternal serology is consistent with prior immunity.

### Limitations

A limitation of this case is the absence of molecular testing (PCR) during pregnancy, as prenatal care occurred at another facility and no diagnostic suspicion was raised. Additionally, the mother was considered immune based on repeated IgG-positive/IgM-negative results—one obtained early in pregnancy and another only near delivery. No serial serologic monitoring was performed throughout gestation. This limited follow-up may have contributed to the absence of laboratory documentation of a potential reinfection or reactivation event. Furthermore, comparative mother–newborn Western blot testing was not performed. Therefore, the newborn screening IgM result obtained during the first days of life should not be interpreted in isolation. In this case, in association with compatible clinical and imaging findings, the diagnosis was supported by the detection of specific anti-*Toxoplasma gondii* IgM at 13 days of life (beyond the 10th day), since IgM detected at birth or during the first days of life may not definitively confirm congenital infection due to the possibility of accidental maternal IgM transfer during delivery. Comparative mother–newborn Western blot testing would have been necessary to distinguish maternal antibodies from those synthesized by the newborn.

## CONCLUSION

Immunity to toxoplasmosis during pregnancy requires reevaluation. Specific preventive counseling should be reinforced for all pregnant women, including those previously infected. Recommended measures include avoiding raw or undercooked meat, thoroughly washing fruits and vegetables, avoiding contact with cat feces and contaminated soil, and following safe food-handling practices. Expanding preventive guidelines and maintaining vigilance throughout prenatal care are crucial to reducing the burden of congenital toxoplasmosis and its sequelae.

## Data Availability

The anonymized dataset generated during this study is available from the corresponding author upon reasonable request.
